# Progress in State Medicine

**Published:** 1898-01-15

**Authors:** 


### PROGRESS IN STATE MEDICINE.

### MARINE HYGIENE.

In his Milroy Lectures,[1] Dr. Collingridge deals with the subject of "Quarantine." The first Quarantine Act in England was passed in the ninth year of the reign of Queen Anne, its object being to prevent the spread to this country of the plague which was then raging in the Baltic. This Act was only temporary, and the period extending from the time of its inception to the repeal of the Quarantine Act in 1896 is marked by numerous similar legislative enactments. Altered views with regard to the infectious nature of plague, and the failure of quarantine regulations to check the progress of cholera, gradually led to increased importance being attached to medical inspection, and in 1874 the International Conference of Vienna recommended the abolition of the older system. This recommendation, however, could accomplish little, as certain of the European Powers dissented. The second conference, held at Dresden, in 1893, was more successful, and was practically the deathblow to quarantine. Indeed, by the new provisions, persons arriving in infected ships are not detained at all, but their names and addresses forwarded to the sanitary authorities of the district to which they are about to proceed. The Quarantine Act thus rendered useless was abolished in 1896, special powers to deal with cholera, yellow fever, and plague being at the same time conferred upon the Local Government Board. The arguments against quarantine are summed up by Dr. Collingridge as follows: (1) The system is based upon a theory that is no longer tenable, viz., that cholera, yellow fever, and plague are contagious diseases; (2) that when carried out as strictly as possible it has not the effect of arresting the importation of disease; (3) that it has never been found possible to maintain quarantine absolutely, and that consequently a country practising it has been subjected to a barbarous, unnecessary, and costly system without even the satisfaction of knowing that the experiment was complete; and (4) that sanitation and a rational system of medical inspection have proved more efficacious, and at the same time less burdensome.

Borthwick[2] criticises unfavourably the system of "limited quarantine" in force in Australia. Under this system, infected ships are detained, cleansed, and disinfected, the sick are isolated, and the apparently well suffer a period of detention varying according to the incubation period of the particular disease. As a matter of fact, no proper means of disinfecting ship, cargo, or clothing exists, nor is there good sanitary accommodation for those detained, or provision of sufficient medical attendance or vaccination. The isolation of the sick is, too, performed in the most perfunctory manner. The cost to the shipping companies for each vessel quarantined is between two and three thousand pounds. As might be expected, the existence of such regulations has led to the concealment of cases of disease. In eight out of sixteen vessels infected with small-pox, the disease was either missed altogether or diagnosed as chicken-pox. The diagnosis having been subsequently established without doubt, it has been found impossible to trace the passengers after landing. Moreover, quarantine has failed to stop the introduction of disease, for it may be imported overland, or by persons coming from short sea voyages, sufficient time for the appearance of symptoms not having elapsed. Land quarantine is a most expensive procedure, seven cases of small-pox occasioning an outlay of nearly £2,000. The adoption of a system of medical inspection would lead to the more ready notification of all cases of sickness, would render indispensable the establishment of isolation hospitals, disinfection apparatus, and staff, and would free traders from very great inconvenience.

The eyesight of seamen is a matter recently reported upon by a special commission of the British Medical Association.[3] Neither in the case of pilots, able seamen, or apprentices is any compulsory official test made for colour blindness or defective sight, thus exposing the public to grave peril and the individual to possible injustice. As the matter stands at present the Board of Trade has no statutory power to enforce qualifications of any kind until the seaman presents himself for examination as second mate. There is, it is true, a voluntary colour and form examination, for which a small fee has to be paid, but this is little used, and its value is discounted by the fact that the test is conducted by persons other than properly qualified ophthalmic surgeons. The tests, too, are avowedly of the simplest possible character, instead of being efficient and searching. Candidates whose eyesight is found to be defective at the second mates' examination are allowed the right of appeal to London. This entails a serious waste of time, and expenditure of money, which would be obviated were the examiners at all centres chosen for their expert knowledge of ophthalmic work. That the tests are used in a very rough and ready manner is proved by the fact that 38 per cent. of those who were rejected and who appealed were, on appeal, passed. Again, the Board of Trade report for 1897 shows that during the previous year, of all persons examined, only 1·02 per cent. were shown to be colour blind, whereas the known percentage of colour blind persons in the community is 4. It is absolutely essential that colour and form tests shall be applied at the threshold of the seaman's career, and that such examination shall be conducted by ophthalmic experts. It is believed that the Board of Trade could, by obtaining an order in Council, acquire power to enforce such a regulation.

Attention has been drawn[4] to the dangers arising from defective jointing of soil pipes from deck closets, when such pipes traverse the lower decks or cargo spaces. The danger is a double one; goods may be damaged from the entrance of sea water through defects in these pipes, and if cases of cholera, dysentery, or enteric fever exist on board, a leaky pipe may be the means of infecting goods in the hold, and thus lead to the spread of disease. The warm, close, and moist atmosphere of the hold is most favourable to the growth of the bacteria of cholera or other excremental diseases. The obvious and simple remedy is that all soil pipes should be carried at once through the ship's side and end high above the water-line. The absolute necessity of keeping the lower decks and cargo spaces in as dry and well-aired a condition as possible has been emphasised by the researches of Sanarelli[5] into the life history of the bacillus of yellow fever. He found the growth of the organism to be markedly encouraged by the presence in the culture medium of certain moulds, it being, indeed, difficult to secure typical cultures of the bacillus in the absence of the parasite. He thus concludes that the damp and mouldy ship's hold is the natural habitat and breeding ground of the bacillus of yellow fever, and where once established it is most difficult to dislodge. Old vessels, with dark, ill-ventilated, damp, and heated holds, are especially favourable to the growth of Bacillus icteroides, and should never be employed in infected zones.

The Committee on Steamship and Steamboat Sanitation of the American Public Health Association[6] reports on the necessity of more attention being paid to passengers' bedding. Linen must be frequently washed; blankets, mattresses, and pillows aired and exposed to sunshine; dining-rooms should be above the water-line, so as to ensure cleanliness, ventilation, and general wholesomeness, and saloons and smoking-rooms require more frequent ventilation and less heating. Every precaution should be taken to prevent the spread of consumption by dried sputa, spittoons being more freely provided, and charged with some disinfecting solution.

1 Lancet, March 13, 1897. 2 Australian Medical Gazette, June 22, 1897. 3 British Medical Journal, Feb. 6, 1897. 4 Practitioner, Nov. 1897. 5 Annales de L'Institut Pasteur, Sept. 25, 1897. 6 Medical Record, Nov. 13, 1897.

